# Spontaneous and stimulated electron–photon interactions in nanoscale plasmonic near fields

**DOI:** 10.1038/s41377-021-00511-y

**Published:** 2021-04-15

**Authors:** Matthias Liebtrau, Murat Sivis, Armin Feist, Hugo Lourenço-Martins, Nicolas Pazos-Pérez, Ramon A. Alvarez-Puebla, F. Javier García de Abajo, Albert Polman, Claus Ropers

**Affiliations:** 1 Center for Nanophotonics, AMOLF, 1098 XG Amsterdam, The Netherlands; 2 4th Physical Institute–Solids and Nanostructures, University of Göttingen, 37077 Göttingen, Germany; 3 Max Plank Institute for Biophysical Chemistry, 37077 Göttingen, Germany; 4 Department of Physical Chemistry and EMaS, Universitat Rovira i Virgili, 43007 Tarragona, Spain; 5 ICREA–Institució Catalana de Recerca i Estudis Avançats, 08010 Barcelona, Spain; 6 ICFO–Institut de Ciencies Fotoniques, The Barcelona Institute of Science and Technology, 08860 Castelldefels (Barcelona), Spain

**Keywords:** Nanophotonics and plasmonics, Nanoparticles

## Abstract

The interplay between free electrons, light, and matter offers unique prospects for space, time, and energy resolved optical material characterization, structured light generation, and quantum information processing. Here, we study the nanoscale features of spontaneous and stimulated electron–photon interactions mediated by localized surface plasmon resonances at the tips of a gold nanostar using electron energy-loss spectroscopy (EELS), cathodoluminescence spectroscopy (CL), and photon-induced near-field electron microscopy (PINEM). Supported by numerical electromagnetic boundary-element method (BEM) calculations, we show that the different coupling mechanisms probed by EELS, CL, and PINEM feature the same spatial dependence on the electric field distribution of the tip modes. However, the electron–photon interaction strength is found to vary with the incident electron velocity, as determined by the spatial Fourier transform of the electric near-field component parallel to the electron trajectory. For the tightly confined plasmonic tip resonances, our calculations suggest an optimum coupling velocity at electron energies as low as a few keV. Our results are discussed in the context of more complex geometries supporting multiple modes with spatial and spectral overlap. We provide fundamental insights into spontaneous and stimulated electron-light-matter interactions with key implications for research on (quantum) coherent optical phenomena at the nanoscale.

## Introduction

Nanoscale optical components enable light manipulation at deep-subwavelength length scales with a broad variety of applications in quantum information systems, optical signal processing, photovoltaics, molecular sensing, chemical catalysis, and more^1^. The small feature sizes rendering the unique optical properties of these structures demand novel optical characterization techniques that overcome the diffraction-limited resolution of traditional light microscopy. In recent years, high-energy electrons (1–300 keV) have been established as a powerful tool to probe optical material properties with extreme spatial, temporal, and energy resolutions^2–4^.

When a swift electron passes through or close to a specimen, its time-varying evanescent electric field polarizes the material for a fraction of a femtosecond, corresponding to an excitation energy spectrum with significant weights between zero and several tens of electron volts^5^. The electron thus provides a unique source of optical material excitations at frequencies within the entire ultraviolet-visible-near-infrared (UV-VIS-NIR) spectral range. The energy transfer during this interaction can be measured experimentally using electron energy-loss spectroscopy (EELS)^3,4^. In addition, cathodoluminescence (CL) spectroscopy enables optical detection of the induced radiative polarization states in the far field^3,4^. Although the excitation process is spontaneous in nature, the light emitted by an optical resonance carries a fixed phase with respect to the electron field, distinguishing it from incoherent light emission upon inelastic electron scattering inside a material^2^. Notably, the measured electron energy-loss and photon-emission probabilities are closely linked to the full and radiative electromagnetic local density of states (EMLDOS), respectively^6,7^. Thus, EELS is sensitive to both the dark and bright modes in a material, while CL unveils the bright modes only^8^. As extensively shown in the past, the two techniques are ideally suited for the correlated structural and optical characterization of plasmonic and dielectric nanoparticles^9–14^, optical waveguides^15–17^, photonic crystal cavities^18,19^, and more^3,4,20^.

Recently, EELS and CL have been complemented with photon-induced near-field electron microscopy (PINEM)^21^. In this technique, swift electrons are used to probe the near field of a material illuminated by an intense laser. While passing through this near field, the electrons undergo one or multiple energy-gain and energy-loss transitions by stimulated absorption and emission of photons at the laser frequency *ω*_L_^22–24^. As a consequence, the initial electron energy spectrum is expanded with sidebands, evenly spaced by the photon energy $\hbar \omega _{\mathrm{L}}$. The population of these sidebands varies with the near-field integral along the electron trajectory and the statistics of the incident light^25^, enabling spatially resolved near-field measurements^26–32^ with fs- and as-temporal^31,33^ and meV-spectral resolutions^34^. The key to the PINEM mechanism is the fact that the evanescent near field provides spatial Fourier components with sufficiently large momenta to bridge the phase mismatch between the electron field and the optical pump field in free space. For large incident light intensities, the PINEM interaction can be a highly efficient process in which nearly every electron undergoes stimulated energy-gain or energy-loss transitions^27^, even leading to hundreds of net photon exchanges^32,35^. In contrast, in EELS and CL, the probabilities for the spontaneous excitation of an optical resonance are comparatively small, typically on the order of 10^−5^–10^−3^ per eV energy bandwidth^2^.

The full exploitation of the rich new physics that the PINEM effect offers is just starting^3,4,25–38^. Recent work has focused on studying the quantum nature of the electron during its interaction with an optical near field and the subsequent modulation of the electron wave packet, enabling exciting phenomena such as the generation of coherent attosecond electron pulse trains^33,36,37^ or electron vortex beams^38^. The relation between the stimulated and spontaneous interaction mechanisms governing PINEM, EELS, and CL has been addressed theoretically for small particles with dipolar resonances^39,40^. However, an experimental comparison of the three techniques on the exact same physical structure has not been reported to date.

In this article, we present spatially resolved EELS, CL, and PINEM measurements in the near field of a single chemically synthesized Au nanostar composed of an ~50-nm-diameter spherical core and sharp conical protrusions with a tip radius of curvature <3 nm^41^. As shown in previous works^42–51^, these tips sustain distinct plasmonic resonances in the VIS-NIR spectral range that give rise to highly confined optical near fields at the tip apexes, providing an ideal geometry to compare EELS, CL, and PINEM measurements at the nanometer length scale. Supported by theoretical considerations and numerical electromagnetic boundary-element method (BEM) calculations, we study the spectral and spatial dependence of the spontaneous electron energy-loss and photon-emission probabilities probed by EELS and CL, respectively, and the stimulated electron-near-field coupling strength measured in PINEM. We discuss their dependence on the electron velocity and link it to the spatial Fourier composition of the optical field in the direction along the electron trajectory. Our findings provide detailed insights into the correlations between spontaneous and stimulated electron–photon interactions, illuminating the link between EELS, CL, and PINEM as a complementary set of techniques in research on quantum coherent optical phenomena at the nanoscale.

## Results

### Theoretical analysis of EELS, CL, and PINEM

First, let us consider the interaction of a swift electron with a simplified model geometry consisting of a single Au tip attached to a spherical core. At an incident energy *E*_0_, we assume that the electron propagates along the **z** direction near the tip apex oriented along the **x** direction. In this configuration, the time-varying evanescent electric field of the electron couples most efficiently to the dominant dipole moment **p**_*x*_ along the symmetry axis of the tip. The *z* component of the induced electric field associated with that **p**_*x*_ dipole acts back on the electron, resulting in an energy loss Δ*E* with a spectral probability distribution peaking around the tip resonance energy $\hbar \omega _0$. Subsequently, the energy transferred to the particle is either dissipated as heat or radiated into the far-field, giving rise to CL.

**Fig. 1:**
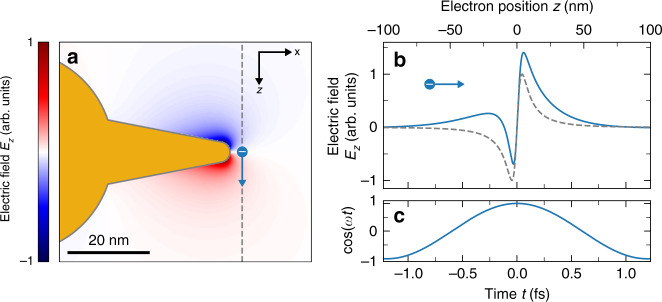
Electron-near-field interaction. BEM calculation of swift electron excitation of a conical Au nanotip attached to a spherical core (tip length 30 nm, cone aperture angle 22° tip apex radius-of-curvature 2.5 nm, core diameter 50 nm). At an incident energy of 20 keV, the electron passes 3 nm away from the tip apex along the dashed line perpendicular to the symmetry axis of the tip (*z* direction). **a** Frozen-time snapshot at a minimum electron-apex distance (*t* = 0) of the *z* component of the electric field induced upon excitation of the tip resonance at $\hbar \omega _0 =$1.73 eV. **b** Dashed-gray curve: field profile along the electron trajectory in **a**, with *z* = 0 corresponding to the tip symmetry axis. Solid-blue curve: time evolution of the field as effectively experienced by the electron. **c** Time evolution of the optical phase of the induced field oscillating at the plasmon resonance frequency $\omega _0$

Figure 1a shows a numerical electromagnetic boundary-element method (BEM)^52–54^ calculation of the *z* projection of the electric field induced by a 20 keV electron incident from the top and traveling along the dashed-gray line (see “Methods” section). We plot the spectral field component induced at the tip resonance frequency *ω*_0_ (corresponding to an energy of $\hbar \omega _0 =$ 1.73 eV) for the moment of maximum approach between the electron and the tip apex, i.e., *t* = 0. As expected for a dipolar mode, the field distribution is strongly localized near the tip apex, vanishing along the symmetry axis of the tip while showing opposite signs above and below (upward field orientation in red; downward field in blue). For a qualitative discussion, neglecting weak coupling to the nanostar core, we consider the spatial distribution of the electron-induced field **E**^e^ to be dominated by the mode profile of the tip resonance, with an associated mode electric field $\vec{\mathcal{E}}$. In the nonrecoil approximation (i.e., ${{\Delta }}E \ll E_0$), we can then write the EELS and CL emission probabilities per unit frequency *ω* as

1 $${{\Gamma }}_{{\mathrm{EELS}}}({\mathbf{R}},\omega ) \approx \frac{{e^2}}{{\pi \hbar }}{\mathrm{Im}}\left\{ {f(\omega )} \right\}\left| {{\int} {{\mathrm{d}}z} \,{\mathcal{E}}_z\left( {{\mathbf{R}},z} \right)\,e^{ - i\frac{\omega }{v}z}} \right|^2$$

2 $${{\Gamma }}_{{\mathrm{CL}}}({\mathbf{R}},\omega ) \approx \frac{{2e^2\omega ^3}}{{3\pi \hbar c^3}}A\left| {f\left( \omega \right)} \right|^2\left| {{\int} {{\mathrm{d}}z} \,{\mathcal{E}}_z\left( {{\mathbf{R}},z} \right)\,e^{ - i\frac{\omega }{v}z}} \right|^2$$

where ${\mathbf{R}} = (x,y)$ and *z* denote the lateral and along-the-beam electron positions, respectively, *e* is the electron charge, *v* is the electron velocity, *c* is the speed of light in vacuum, *f*(*ω*) is a shape- and material-dependent polarization function, and *A* describes the radiation efficiency of the mode (e.g., *A* = 1 for a dipolar mode). The exponential term in the integral expression governing Eqs. (1) and (2) describes temporal oscillations in the phase of the field as the electron passes by the tip apex at a constant velocity *v* (time-position relation *t* = *z*/*v*). The solid-blue curve in Fig. 1b shows the full excursion of the real part of the field experienced by the electron along its trajectory. For reference, the dashed-gray curve shows a cross section through the field distribution in Fig. 1a at *t* = 0, and the time evolution of its optical phase is plotted in Fig. 1c. Classically, we can say that the electron experiences subsequent acceleration and deceleration along its trajectory, leading to alternating positive and negative contributions to the interaction probability. As a result, ${{\Gamma }}_{{\mathrm{CL}}}$ and ${{\Gamma }}_{{\mathrm{EELS}}}$ depend only on the Fourier amplitude of ${\mathcal{E}}_z$ at a spatial frequency $q = \omega /v$, corresponding to a wave that propagates in phase with the electron throughout the interaction. Incidentally, in the limit of large velocity *v*, the electron couples less efficiently to the evanescent electric field components of the tip mode. The integrand in Eqs. (1) and (2) then becomes closer to anti-symmetric with respect to *z* = 0 (*t* = 0), and its net integral is reduced (i.e., the solid-blue curve approaches the shape of the dashed-gray curve in Fig. 1b).

Let us now consider the effect of an optical pump field that is polarized along the symmetry axis and tuned to the resonance frequency of the tip. For typical illumination intensities on the order of hundreds of MW cm^−2^, light induces a much larger dipole moment than that generated by an individual electron. As a result, the interaction probability is strongly enhanced at the pump field frequency *ω*_L_, facilitating both electron energy-gain and energy-loss transitions by stimulated absorption or emission of photons at an energy exchange $\hbar \omega _{\mathrm{L}}$. As demonstrated in ref. ^27^, the energy spectrum of the transmitted electrons then evolves into a ladder of quantum coherent energy-gain and -loss states, with the population of the ladder states governed by Rabi oscillations in the electron-light energy exchange process.

The probability that an electron undergoes a net amount of *n* stimulated energy-gain or -loss transitions can be described by Bessel functions of the first kind, *n*th order^23,27^

3 $$P_n\left( {{\mathbf{R}},\omega } \right) = J_n^2\left( {2\left| {\beta \left( {{\mathbf{R}},\omega } \right)} \right|} \right)\,\delta (\omega - \omega _{\mathrm{L}})$$

where *β* is the coupling coefficient of the electron to the time-varying laser-induced electric field $2{\mathrm{Re}}\left\{ {{\mathbf{E}}^{\mathrm{L}}e^{ - i\omega _{\mathrm{L}}t}} \right\}$. Again, assuming the field distribution to be dominated by the tip resonance, we can write^25,37^

4 $$\left| {\beta \left( {{\mathbf{R}},\omega } \right)} \right| \approx \frac{e}{{\hbar \omega }}\sqrt {\eta I} \left| {f\left( \omega \right)} \right|\left| {{\int} {{\mathrm{dz}}} \,{\mathcal{E}}_z\left( {{\mathbf{R}},z} \right)\,e^{ - i\frac{\omega }{v}z}} \right|$$

where *η* is the coupling efficiency of the pump field to the tip resonance for a given angle of incidence and polarization, and *I* is the incident field intensity. Incidentally, it has been rigorously shown^25^ that the coupling coefficient associated with a mode depends on its population *n* as $\left| \beta \right| \propto \sqrt n$, which again corroborates the dependence shown in Eq. (4) on efficiency and intensity because $n \propto \eta I$. We note that the coupling coefficient *β* is denoted as *g* elsewhere^27,32^, accompanied by a leading factor of 1/2 and a different normalization of the time-varying field as ${\mathrm{Re}}\left\{ {{\mathbf{E}}^{\mathrm{L}}e^{ - i\omega _{\mathrm{L}}t}} \right\}$, without the leading factor of 2. Equations (1), (2), and (4) show that for a fixed electron energy and in the limit of an isolated tip mode, the square of the coupling strength $\left| \beta \right|^2$ has the same spatial dependence as the spontaneous electron energy-loss and photon-emission probability densities ${{\Gamma }}_{{\mathrm{CL}}}$ and ${{\Gamma }}_{{\mathrm{EELS}}}$. Furthermore, $\left| \beta \right|^2$ equally scales with $\left| {f\left( \omega \right)} \right|^2$ as ${{\Gamma }}_{{\mathrm{CL}}}$, demonstrating that both PINEM and CL depend on the radiative nature of the tip modes.

### EELS and CL experiments

**Fig. 2:**
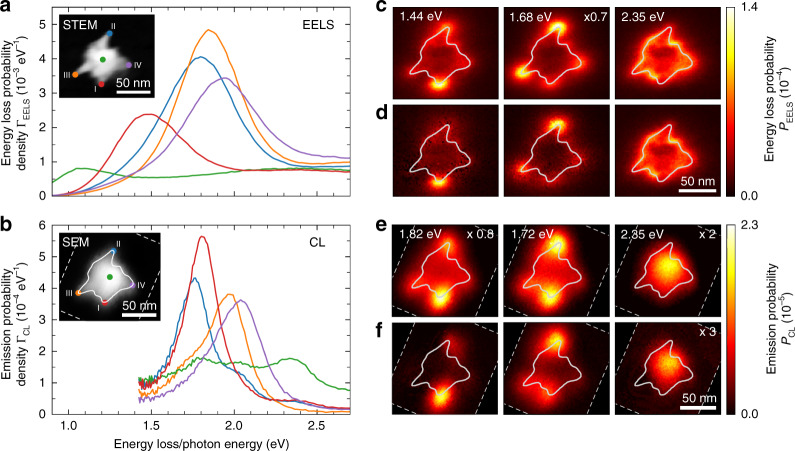
EELS and CL measurements. **a** 200 keV STEM-EELS and **b** 20 keV SEM-CL spectra taken at four different tips and at the core of the same chemically synthesized Au nanostar. Electron beam positions are indicated by the color-matched dots in the insets, showing a STEM bright-field image and an SEM image of the nanostar. All spectra represent an average taken over 5 × 5 neighboring pixels. Energy-filtered **c** EELS and **e** CL probability distributions obtained for a bandwidth of ±25 meV around the plasmon resonance energies indicated on the top. Fitted **d** EELS and **f** CL probability distributions, revealing the extracted contribution of the tip plasmon resonances to the raw energy-filtered maps in **c** and **e**. The acquisition boundaries of the CL data set are indicated by the thin dashed lines in **b**, **e**, and **f**. The solid lines superimposed on the EELS and CL maps and the SEM image illustrate the approximate contour of the nanostar as inferred from the STEM bright-field image in **a**

Spatially resolved EELS and CL measurements were performed in a scanning transmission electron microscope (STEM) and a scanning electron microscope (SEM) operating at acceleration voltages of 200 kV and 20 kV, respectively. STEM and SEM images of the nanostar taken during data acquisition are shown in the insets in Fig. 2a, b. The electron beam was raster-scanned over the sample along a two-dimensional grid of pixels with dimensions of (2×2) nm^2^. A 30-nm-thin silicon nitride support membrane was used to ensure minimum inelastic scattering of the transmitted electrons. Figure 2a shows the EELS spectra acquired at four different tip apexes and the nanostar core. Note that the spectra are numerically deconvolved by the contribution of the zero-loss peak (ZLP), yielding a substantial gain in energy resolution for a non-monochromated electron source (see “Methods” section)^55^. At the tips, we observe plasmonic resonances that give rise to pronounced maxima at 1.48, 1.80, 1.84, and 1.94 eV, with a full-width-at-half-maximum (FWHM) on the order of 400 meV. The core shows a broad, comparably flat spectrum, with another faint peak near 1.0 eV. Figure 2b shows the CL spectra acquired at approximately the same five positions. Again, the tip spectra indicate distinct plasmonic resonances, with maxima peaking at energies of 1.76, 1.80, 1.97, and 2.04 eV and an FWHM of ~200 meV. In the core spectrum, we observe peaks of similar width, yet smaller amplitude, at 1.8, 2.0, and 2.4 eV.

As is evident from Fig. 2a, b, there is significant spectral overlap between the features associated with the different tips and the nanostar core. The high spectral resolution in CL permits the observation of shoulders on the low- or high-energy side of the peaks, suggesting weak coupling between particular tip resonances. In good correspondence with the results of earlier experiments on Au nanostars^47,49,50^, we attribute the high-energy peak near 2.4 eV in CL to the plasmon resonance of the nanostar core. The side peaks in the core spectrum at 1.8 and 2.0 eV indicate coupling between the core and tip modes, as suggested in ref. ^44^. In EELS, multiple inelastic scattering losses hinder the observation of the core resonance, also giving rise to a low-energy feature near 1.0 eV that we identify as a noise artefact of the applied ZLP deconvolution algorithm^55^.

To disentangle the spectral and spatial contributions of individual plasmon resonances, we fit our data with a model assuming a dominant contribution by the resonances of the four tips labeled by Roman numerals I-IV in the insets in Fig. 2a, b. In CL, we also take into account the core resonance. In this approach, we neglect retardation effects, assuming that the modes have vanishing spectral or spatial overlap^7^. In the past, a similar procedure has been applied to nanostars^47^, nanotriangles^8^, and branched nanostructures^56^. Further details on our analysis procedure are given in the “Methods” section.

An overview of the derived resonance energies and linewidths is given in Table 1. The linewidths retrieved from the CL analysis range between 0.16 and 0.27 eV, while those obtained from the EELS data range between 0.33 and 0.44 eV. Contrasting these values indicates residual spectral broadening in EELS due to the relatively large initial energy spread of the electron beam. Comparing the resonance energies for tips II–IV, we find minor blue shifts of 2–5% in CL compared to EELS, while for tip I, we notice a considerable blue shift of 26%. Earlier work on silver nanotriangles has demonstrated spectral shifts between EELS and CL due to dissipation in the particle itself and the support substrate^8,57^. However, we note that our EELS and PINEM measurements were taken first, followed by gentle O_2_-plasma treatment of the sample. This procedure was required to reduce signal degradation and accompanying spectral blue shifts during prolonged CL acquisition (20–30 min). Therefore, we assume that contamination with residual chemicals from the synthesis procedure and their reaction to the electron beam are primarily responsible for the discrepancies between the EELS and CL responses of tips II–IV. Additionally, the tips might have slightly deformed as a result of oxygen bombardment or laser-induced heating during PINEM acquisition, most likely explaining the substantial spectral shift of tip I. Indeed, the tip resonances are highly sensitive to the exact tip morphology, with decreasing sharpness and aspect ratio resulting in blue shifts of tens to hundreds of meV^41^.

**Table. 1:** Fitted plasmon resonance energies (*E*_0_) and FWHM linewidths (*γ*) derived from the EELS and CL data (Fig. 2a, b)

	EELS (200 keV)		CL (20 keV)	
	*E*_0_ (eV)	*γ* (eV)	*E*_0_ (eV)	*γ* (eV)
Tip I (red)	1.44	0.40	1.82	0.16
Tip II (blue)	1.68	0.36	1.72	0.27
Tip III (orange)	1.85	0.33	1.95	0.22
Tip IV (purple)	1.98	0.44	2.06	0.24
Core (green)	–	–	2.35	0.25

In Fig. 2c, e, we show energy-filtered EELS and CL maps integrated over a bandwidth of ±25 meV around the resonance energies of tips I and II, as determined from the EELS and CL spectra, respectively, as well as the core resonance (see labels on the top left of each panel). In the latter case, the core region lights up in CL, while it remains mostly dark in EELS. The tip resonances are clearly observed by both techniques, giving rise to strong interaction maxima near the tip apexes. However, as a consequence of spectral overlap between the modes, we can observe multiple tips to light up for given resonance energy. Therefore, in Fig. 2d, f, we show the amplitude distributions retrieved from the spectral analysis of our data, resolving the ambiguities in the raw energy-filtered maps. For reference, we also plot the contribution of a simple background model to the EELS signal assuming a combination of plasmonic and inelastic scattering losses around 2.35 eV.

### PINEM experiments

**Fig. 3:**
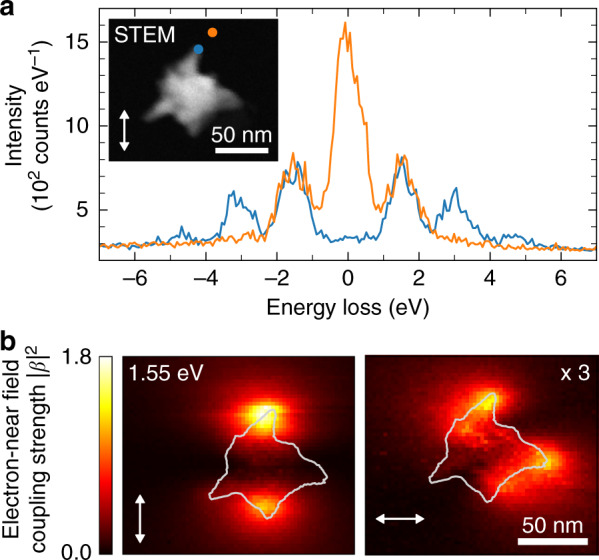
PINEM measurements. **a** 200 keV STEM-PINEM spectra of a Au nanostar corresponding to regions of strong (blue curve) and weak (orange curve) electron-near field coupling. The electron beam positions are indicated by the color-matched dots in the inset, showing an HAADF image of the nanostar. The white double arrow represents the approximate in-plane polarization of the driving field. **b** Maps of the electron-near-field coupling strength derived from the energy spectra of the transmitted electrons for two orthogonal laser polarizations, as indicated by the white double arrows. The solid lines illustrate the approximate contour of the nanostar, as inferred from the HAADF image in **a**. Intensities in the right-hand panel have been scaled by a factor of 3

Spatially resolved PINEM measurements were performed in the same STEM instrument as used for EELS experiments at an acceleration voltage of 200 kV. The instrument was operated in a laser-triggered ultra-fast photoemission configuration in which sub-picosecond electron pulses were temporally synchronized with 3.4-ps optical pump pulses of 1.55 eV central photon energy. The light was incident near normal to the sample plane and had a peak intensity on the order of 1 GW cm^−2^. As in EELS and CL, the pulsed electron beam was raster-scanned over the sample along a two-dimensional grid of pixels with dimensions of (2×2) nm^2^. The inset in Fig. 3a shows a STEM high-angle-annular-dark-field (HAADF) image of the nanostar prior to PINEM acquisition, with the white arrow indicating the laser polarization. The main panel in Fig. 3a shows PINEM spectra taken near the apex of tip II with approximate resonance energy of 1.7 eV (as determined by EELS and CL) at distances of ~5 nm (blue) and ∼20 nm (orange) from the tip. At a 20 nm distance, we observe a pronounced ZLP accompanied by first-order emission and absorption peaks ($\pm \hbar \omega _{\mathrm{L}}$) clearly visible on both the energy-gain and energy-loss sides of the spectrum. In contrast, at a 5 nm distance, the ZLP is fully depleted, and the first-, second-, and third-order sidebands ($\pm n\hbar \omega _{\mathrm{L}}$ for *n* = 1, 2, and 3) are observed. This trend indicates increasing electron-near-field coupling with decreasing separation from the tip apex. Each sideband and the ZLP have an FWHM of ~0.9 eV, as primarily determined by the energy spread of the electron pulses. The latter again follows mainly from excess energy in the photoemission process (source excitation photon energy 3.1 eV) and space charge-related broadening of the electron energy distribution close to the tip emitter^58,59^. However, we note that a fundamental limit to the spectral resolution in PINEM is only imposed by the bandwidth of the employed laser system^34^.

To map out the laser-induced optical field, we derive the electron-near-field coupling constant |$\beta |$ from the PINEM spectrum recorded at every electron beam position. To this end, we approximate the electron energy distribution by a comb of 2*N* + 1 pseudo-Voigt profiles that are spaced by the photon energy $\hbar \omega _{\mathrm{L}}$, each resembling the approximate line shape of the ZLP. The integral of the *n*th profile is then determined by the occupation probability *P*_*n*_ of the *n*th energy-gain and -loss sideband, which again follows from the local coupling constant |$\beta |$, as shown in Eq. (3) (see “Methods” section). On the left in Fig. 3b, we plot the spatial distribution of the squared electron-near-field coupling strength $\left| \beta \right|^2$ derived for the same laser polarization as shown in Fig. 3a. Similar spatial distributions to those obtained by EELS and CL measurements are observed near the apexes of tips I and II, which are both approximately aligned with the laser polarization. Taking the EELS measurements as a reference, this agrees well with the fact that the corresponding resonance energies are closest to the central pump photon energy of 1.55 eV. Indeed, tip III, with its resonance further to the blue, shows almost no response although its symmetry axis is nearly aligned with that of tip II. To verify the correlation with the tip orientation, the right panel of Fig. 3b shows a $\left| \beta \right|^2$ map for the orthogonal laser polarization. Clearly, the coupling strength around tips I and II is now strongly reduced (note that the data are scaled by a factor of three), and tip IV, which is better aligned with the polarization, lights up. However, we see that the effect is comparably small as the tip resonance energy is furthest from the central pump photon energy. We note that similar to the polarization, the direction of incidence of the pump field influences the excitation efficiency of a given tip mode. This behavior might contribute to differences in the maximum electron-near-field coupling strength observed between tips I and II.

### BEM calculations

To complement our experimental data, we resort to numerical BEM calculations and further study the EELS, CL, and PINEM responses of our model geometry introduced in Fig. 1. The optical pump field in PINEM is modeled by a monochromatic plane-wave incident from the top and polarized along the symmetry axis of the tip, assuming resonant tip mode excitation at a pump photon energy of 1.73 eV. For the sake of simplicity, the effect of a substrate is not taken into account^49^.

**Fig. 4:**
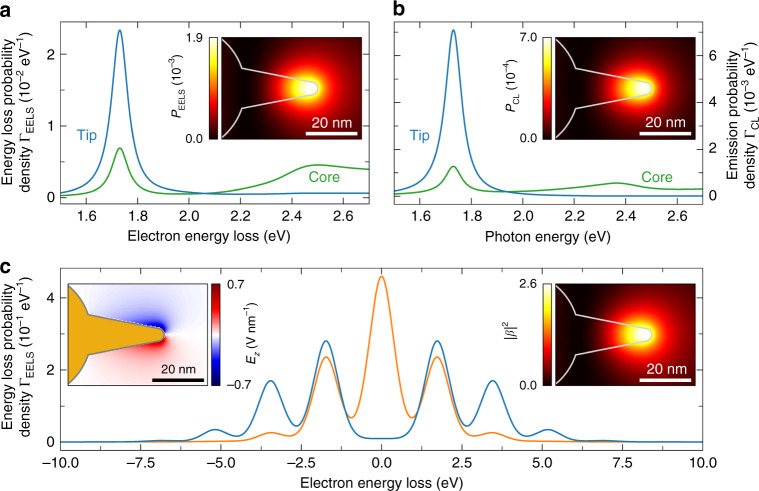
Numerical BEM calculations. BEM calculations of **a** 200 keV EELS and **b** 20 keV CL spectra for electrons passing through the center of the spherical core (green) and 3 nm away from the tip apex (blue). The insets show EELS and CL probability distributions obtained for a spectral bandwidth of ±25 meV around the tip resonance at 1.73 eV. **c** Calculated 200 keV PINEM spectra for electrons with an energy spread of 0.9 eV passing 3 nm (blue) and 20 nm (orange) away from the tip apex (plane wave pump field incident along ${\mathbf{z}}$ and polarized along **x**, $\hbar \omega _{\mathrm{L}}$=1.73 eV photon energy, and 0.02 GW cm^−2^ intensity). The left inset shows a time snapshot of the *z* component of the optically induced near field in the *x*–*z*-symmetry plane of the tip; the right inset shows a *x*–*y* map of the calculated squared electron–photon coupling strength $\left| \beta \right|^2$

Figure 4 shows 200 keV EELS (a) and 20 keV CL (b) spectra calculated for an electron passing through the center of the spherical core (green curves) and 3 nm away from the tip apex (blue curves). For the electron passing near the tip, the tip resonance is clearly observed as a sharp maximum at 1.73 eV with a spectral linewidth of ~100 meV (FWHM). In the core spectra, the core resonance appears as a broader maximum near 2.4 eV, with the small peak at 1.73 eV indicating weak coupling to the tip resonance, in good agreement with the experiments. The insets show the *x*–*y* EELS and CL distributions at ~1.73 eV (bandwidth ±25 meV), revealing a strong interaction maximum near the tip apex. In Fig. 4c, we show 200 keV PINEM spectra calculated for the electron passing by the tip apex at distances of 3 nm (blue curve) and 20 nm (orange curve). For the larger distance, a pronounced ZLP and only the first-order energy-gain and -loss sidebands $(\pm \hbar \omega _{\mathrm{L}})$ are observed, while closer to the tip, the ZLP is fully depleted, and the first-, second-, and third-order sidebands ($\pm n\hbar \omega _{\mathrm{L}}$ for $n = 1,2,3$) can be seen. In the calculations, a light intensity of 0.02 GW cm^−2^ is chosen to best match the electron energy modulation observed in the experiments. The inset on the left shows the calculated *z* component of the plane wave-induced near field ${\mathbf{E}}_{\mathrm{z}}^{\mathrm{L}}$ in the *x*–*z* symmetry plane of the tip. We find a similar spatial distribution as for the electron-induced field ${\mathbf{E}}_z^{\mathrm{e}}$ plotted in Fig. 1a, showing that both distributions are dominated by the electric field profile ${\mathcal{E}} _z$ of the tip mode. In the inset on the right, we plot the *x*–*y* distribution of the squared electron–photon coupling strength $\left| \beta \right|^2$. Upon first inspection, there is a good qualitative agreement between the EELS, CL, and PINEM maps, despite fundamentally different near-field excitation mechanisms.

Comparing the measured and calculated EELS and CL spectra, we consistently find the peak electron energy-loss probability density Γ_EELS_ to exceed the peak photon-emission probability density Γ_CL_ by one order of magnitude (cf. Figs. 2a, b and 4a, b). This observation reflects strong non-radiative losses in the Au plasmonic nanostar at optical frequencies^8^. However, in absolute terms, the measured EELS and CL amplitudes are an order of magnitude lower than those obtained for the model geometry. A plausible reason for this deviation are the approximately two- and four-times larger resonance linewidths retrieved from the EELS and CL data, respectively. Among others, such spectral broadening can result from a higher damping rate in the metal than that calculated from optical constants for an extended Au thin film^60^ as well as substrate effects^49,61^, electron beam-induced carbon contamination or nonlocal effects in the tiny plasmonic nanotips^62^. As discussed above, considerable broadening is further introduced in EELS by the finite energy spread of the electron beam. In CL, we note that light is only collected in the upward hemisphere using a parabolic mirror with a limited collection solid angle. In fact, depending on the precise tip orientation, a significant fraction of the radiation can be emitted towards the substrate, drastically decreasing the collection efficiency^49^. Comparing the measured and calculated PINEM spectra, the latter show a similar electron energy modulation for a one to two orders of magnitude lower pump field intensity. Experimental factors contributing to this deviation can be slight off-resonant tip excitation, reduced coupling efficiency in the presence of a substrate, imperfect laser alignment relative to the tip symmetry axis (polarization/direction of incidence), optical losses upon light injection into the STEM instrument, and larger ohmic damping losses than predicted by the calculations. Incidentally, for off-resonant tip excitation at 1.55 eV, a comparable electron energy modulation is obtained assuming an incident field intensity of 0.25 GW cm^−2^. However, we note that off-resonant excitation more drastically affects the response of the model geometry because the calculated tip resonance happens to be narrower than in the experiments.

### Spatial dependence of EELS, CL, and PINEM

**Fig. 5:**
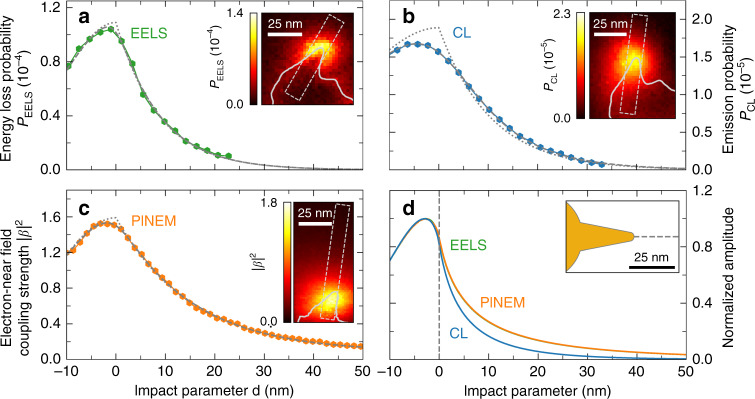
EELS, CL, and PINEM amplitude profiles. Intensity profiles through **a** 200 keV EELS (1.68 eV), **b** 20 keV CL (1.72 eV), and **c** 200 keV PINEM ($\hbar \omega _{\mathrm{L}}$*=* 1.55 eV photon energy) distributions along the symmetry axis of tip II. We also plot model fits assuming Gaussian broadening of the data due to the finite width of the electron beam (dashed curves) and deconvoluted model functions (dotted curves). An impact parameter of *d* = 0 corresponds to the approximate position of the tip apex derived from the fitting procedure. **d** BEM calculations of 200 keV EELS (green, hidden behind the PINEM curve), 20 keV CL (blue), and 200 keV PINEM (orange curve) profiles along the tip symmetry axis of the model geometry. The calculated profiles are normalized to their respective amplitudes at the tip apex. As in the experiments, EELS and CL probabilities are calculated for a spectral bandwidth of ±25 meV around the tip resonance at 1.73 eV, while the PINEM interaction strength is calculated for laser polarization along the symmetry axis of the tip at an excitation energy of $\hbar \omega _{\mathrm{L}}$ *=* 1.73 eV

To study the spatial dependence of our EELS, CL, and PINEM distributions, we plot intensity profiles along the symmetry axis of tip II and the model nanotip in Fig. 5. The experimental profiles are obtained by linearly interpolating and averaging the data within the dashed boxes shown in the insets. The EELS and CL data correspond to the fitted loss and emission probability distributions derived from the measurements presented in Fig. 2 at 1.68 and 1.72 eV, respectively. For PINEM, data were derived from an additional measurement to that shown in Fig. 3b (left) at a higher resolution of (1×1) nm^2^ pixel^−1^. In the calculations, we neglect a vanishing contribution from the core resonance due to its minor spectral overlap with the tip mode (Fig. 4a, b).

To quantitatively compare the measured and calculated profiles, we fit the former with a model assuming an evanescent exponential decay away from the tip apex with a characteristic 1/*e* decay length *δ*^63^. The signal along the tip is described by a half-Gaussian distribution peaking at the tip apex, while the finite width of the electron probe is introduced by convolution with a Gaussian resolution function of standard deviation *σ*. The fitted curves (dashed lines) and deconvoluted model functions (dotted lines) are plotted in Fig. 5a–c. We obtain beam widths of $\sigma _{{\mathrm{CL}}} =$ (5.3 ± 0.2) nm for the CL, $\sigma _{{\mathrm{EELS}}} =$ (1.6 ± 0.5) nm for the EELS, and $\sigma _{{\mathrm{PINEM}}} =$ (2.2 ± 0.3) nm for the PINEM measurements. The characteristic decay lengths are found to be $\delta _{{\mathrm{EELS}}} =$ (8.7 ± 0.3) nm, $\delta _{{\mathrm{CL}}} =$ (10.5 ± 0.2) nm, and $\delta _{{\mathrm{PINEM}}} =$ (15.2 ± 0.2) nm. The BEM calculations yield an identical decay length of 8.1 nm for EELS and PINEM at an electron energy of 200 keV, while a smaller value of 5.5 nm is obtained for CL at an electron energy of 20 keV (relative to the signal amplitude at the tip apex).

In comparison, the measured and calculated profiles show a very similar functional shape, with the maximum coupling strength occurring at or a few nanometers inwards from the tip apex. This result is in good agreement with the maxima observed in the electron- and laser-induced electric field distributions plotted in Fig. 1a and the left-hand inset in Fig. 4c. Excellent agreement between the measured and calculated decay lengths is found for EELS, while for CL and PINEM, the experimental values show an upwards deviation of almost 50%. Comparing the nanostar dimensions retrieved from our SEM and STEM images, we estimate a length scale calibration error on the order of 10–20%. Additionally, we note that the model nanotip only approximates the actual shape of tip II and thus might have a slightly different mode-field profile. For our SEM-CL instrument, a spatial accuracy limit of 3 nm was found in previous work^63^, which is mostly determined by the comparably large electron beam probe width. In PINEM, an uncertainty of a few nanometers could be introduced by mechanical drift, among others resulting from nanoscale heat expansion under laser beam exposure. Such an effect arises from the relatively long acquisition times that are required to resolve PINEM spectra at low pulsed electron beam currents. Notably, an intensity profile through the measured PINEM distribution in Fig. 3b yields a decay length of ∼10 nm at a larger pixel size (shorter acquisition time per unit area), closer to the calculated value. Deviations between the measured distributions due to slightly different spectral responses in EELS and CL, and off-resonant tip excitation at a photon energy of 1.55 eV in PINEM, are expected to be <10% (corresponding to the relative differences in optical wavelength). However, we note that the laser polarization in PINEM does not perfectly align with the tip axis, which could cause a minor, mostly transverse broadening of the interaction maximum due to contributions of other nearby sharp features to the local field enhancement.

We conclude that within experimental uncertainties of a few nanometers (see above), we find good agreement between the measured EELS, CL, and PINEM profiles. This is in line with Eqs. (1), (2), and (4), suggesting that in the limit of an isolated dipolar tip mode the underlying spontaneous and stimulated electron–photon interaction processes share the same spatial dependence on the modal electric field profile. Furthermore, our BEM calculations confirm that for an electron energy of 200 keV the EELS and PINEM profiles perfectly overlap, despite fundamentally different excitation mechanisms. Interestingly, however, the CL profile calculated for an electron energy of 20 keV decays somewhat faster, as could not be resolved experimentally. Careful inspection of Eqs. (1), (2), and (4) further shows that this subtle deviation follows from the dependence of the electron-near-field interaction strength on the integral of the parallel electric field component ${\mathcal{E}}_z$ along the electron trajectory. As discussed in detail below, electrons of different energies, therefore, probe different Fourier components with different spatial contributions to the optical field.

### Dependence on electron energy

**Fig. 6:**
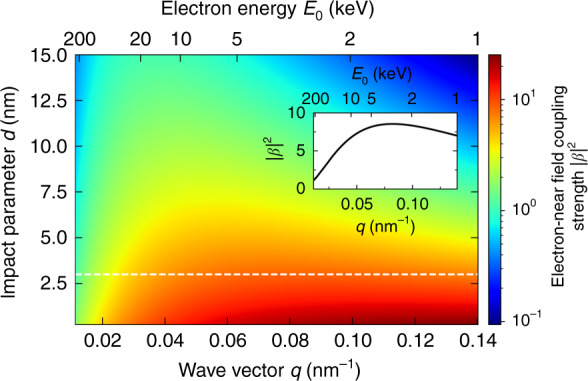
Electron energy dependence of the electron-near-field coupling strength. BEM calculation of the laser-induced electron-near-field coupling strength $\left| \beta \right|^2$ as a function of the along-the-beam wave vector $q = \omega /v$ and impact parameter *d* relative to the tip apex. Data are obtained for a fixed photon energy of $\hbar \omega$=1.73 eV matching the tip resonance energy and for varying electron energies *E*_0_ (see top axis). Other excitation parameters are identical to those in Figs. 4c and 5d. The inset shows a profile along the white dashed line for *d* = 3 nm, representing the local spatial Fourier composition of the optical field along the electron trajectory. For a given *E*_0_, the electron can only couple to the Fourier field component with a spatial phase advance *q*, affecting both the amplitude and the spatial distribution of $\left| \beta \right|^2$

The consequences of the electron energy-dependent near-field coupling are illustrated in Fig. 6. The color map shows the Fourier amplitude of the laser-induced ${\mathbf{E}}_z^{\mathrm{L}}$ distribution plotted in Fig. 4c as a function of impact parameter *d* (i.e., distance away from the tip apex) and along-the-beam wave vector *q* (with the corresponding electron energies *E*_0_ shown on top). The data are expressed in terms of the squared electron–photon interaction strength $\left| \beta \right|^2$.

Two striking trends can be observed in Fig. 6. For any spatial frequency *q*, or equivalently, electron energy *E*_0_, the near-field coupling strength rapidly falls off with impact parameter *d*, and the local maximum gradually shifts towards smaller *q*. We can understand this behavior from the fact that not only the field intensity but also the field confinement monotonically decreases away from the tip apex. For a given beam position, the maximum coupling strength, therefore, occurs at an electron energy for which 2*π*/*q* coincides with the period of the dominant spatial Fourier component in the near field. As an example, the inset shows $\left| \beta \right|^2$ as a function of *q* for an impact parameter of *d* = 3 nm. The maximum coupling occurs for the Fourier component with *q* = 0.08 nm^−1^, which is best matched by an electron with an incident energy *E*_0_ = 3.5 keV. Increasing the distance from the tip, the higher spatial frequency components quickly die out, and the coupling strength peaks for higher electron energy. Importantly, Eqs. (1), (2), and (4) show that this trend is independent of the spontaneous or stimulated nature of the interaction. Incidentally, even a resonantly driven mode that gives rise to an intense near field cannot efficiently exchange energy with an electron when the phase-matching condition addressed above is not fulfilled. This becomes apparent for the largest electron energies in Fig. 6, where the maximum interaction strength near the tip is substantially reduced as compared to the lower electron energies. As discussed earlier, in the limit of large electron velocity, the interaction with evanescent wave components is strongly reduced. Likewise, when the structure is too large to sustain field components with sufficiently large momenta, the interaction strength again drops with decreasing electron energy.

## Discussion

Our conclusions were derived in the limit of small plasmonic nanotips that support a single dipolar mode, yet they can be extended to more generic geometries if the electron-near-field interaction is considered a sum of contributions of distinct eigenmodes that are determined by their spectral and spatial field properties^8,64^. For instance, EELS measurements on a mesoscopic plasmonic taper have shown that the electron selectively interacts with spatially overlapping circumferential modes, depending on the projection of their angular phase pattern along the electron trajectory^65^. Other than in EELS, however, we stress that, in general, the CL and PINEM response of a sample depends on the coherent rather than incoherent superposition (i.e., the amplitude and relative phase) of the modes excited by the electron or the external pump field, respectively^7^. Incidentally, previous work has demonstrated a close relation between CL and optical scattering^8^, underlining the mutual dependence of CL and PINEM on the far-field characteristics of a material. However, we are reminded that the CL signal can involve incoherent light emission resulting from bulk losses inside a material or coherent radiation channels that are not accessible from the far field, such as transition radiation^2^. Additionally, we note that in this work, a classical understanding of CL was adopted that is consistent with a point-like description of the electron^2^. Within the framework of quantum electrodynamics, research is now examining the role of the electron wave nature in its interaction with light, among others addressing the question of coherence transfer from an external reference field to CL by free electrons^66–68^.

Assuming that neither the electron velocity nor its trajectory are altered during the interaction (nonrecoil approximation), our BEM calculations have shown that rather low-energy electrons couple most strongly with the tightly confined optical near fields at the tip apexes. This is a reasonable approximation for spontaneous single-photon exchanges as in EELS and CL, and the moderate stimulated electron energy modulations observed in our PINEM experiments. Importantly, we find our results in good agreement with those of previous comprehensive treatments of the electron-near-field interaction^69–72^. In recent work^69,70^, the strong stimulated coupling between free-electron wave packets and the near field of small nanoparticles has been rigorously discussed beyond the adiabatic regime (i.e., taking into account the recoil upon momentum exchange with a photon). Despite significant transverse diffraction of the electron wave packet, the interaction strength was found to peak at non-relativistic electron energies down to hundreds of eV, with the optimum coupling velocity following phase-matching arguments^69,70^. In other work^71^, the shape-independent maximal spontaneous electron energy-loss and photon-emission probabilities near a dielectric object were studied by considering its interaction with the evanescent electron field as a scattering problem. In close proximity to the sample boundary, the strongest scattering was again found upon interaction with slow non-relativistic electrons, which generate overall larger near-field amplitudes than faster relativistic electrons^71^, also in agreement with previous studies of small plasmonic structures^72^. Practically, however, we note that non-relativistic electron energies are not accessible in STEM, while SEM requires low-energy compatible electron optics to maintain a reasonable beam quality.

In conclusion, we have demonstrated spatially resolved EELS, CL, and PINEM measurements of tightly confined optical near fields at the tip apexes of an Au nanostar, enabling the direct correlation of spontaneous and stimulated electron–photon interactions at the nanometer length scale. In EELS and CL, we observe spontaneous electron-near-field coupling to a number of tip resonances in the VIS-NIR spectral range, while the stimulated interaction in PINEM strongly depends on the polarization of the pump field and its spectral overlap with these modes. We show that all three techniques resolve highly localized interaction maxima at the tip apexes with a lateral spatial extent on the order of 10 nm. Supported by numerical BEM calculations and in agreement with theory, we conclude that in the limit of an isolated dipolar tip mode, spatial variations in the electron–photon interaction are independent of the process being driven by the electron itself (as in EELS and CL) or an external pump field (as in PINEM). Instead, the measured spontaneous and stimulated coupling distributions are fully determined by the modal electric field profile. However, we show that the coupling strength crucially depends on the electron velocity and link this to the spatial Fourier composition of the optical field component parallel to the electron trajectory. Our results contribute to the thorough understanding of electron-light-matter interactions while providing valuable guidelines for the interpretation of further correlative EELS, CL, and PINEM measurements towards new insights in nanophotonics.

## Methods

### Sample preparation

Au nanostars were prepared by modification of a previously reported procedure using a seeded growth approach^41,73^. First, spherical Au seeds of ~12 nm diameter were produced by a modification of the well-known Turkevich method^74^. The seeds were synthesized by the subsequent addition of dehydrated trisodium citrate (C_6_H_5_Na_3_O_7_·2H_2_O, 11 mL, 0.1 M) and gold(III) chloride trihydrate (HAuCl_4_ ∙ 3H_2_O, 833 μL, 0.1 M) to boiling Milli-Q water (500 mL) at intervals of 10 min and under vigorous stirring. After 30 min of boiling, the solution was brought to room temperature, and the particles were added drop-by-drop under stirring to an aqueous polyvinylpyrrolidone (PVP) solution (500 mL, 0.27 mM). Finally, the Au nanoparticles were centrifuged (9000 rpm, 35 min) and dispersed in absolute ethanol (EtOH, 50 mL) to achieve a final Au concentration of 16.2 × 10^−4^ M. Next, Au nanostars were grown by the fast addition of PVP-coated Au seeds in EtOH (350 μL) to a PVP solution in *N*,*N*-dimethylformamide (DMF, 7 g, 35 mL) containing freshly prepared HAuCl_4_ (75 μL, 0.12 M aqueous solution). Within 15 min, the color of the solution turns blue, indicating the formation of Au nanostars. The solution was stirred overnight to ensure the reduction of all reactants. DMF and excess PVP were removed by several centrifugation steps: the first step at 7500 rpm for 40 min followed by four more iterations at 7000 rpm for 10 min each. For each step, the particles were resuspended in EtOH (35 mL). Eventually, Au nanostars (5 μL, 0.8 mM) were deposited on a TEM silicon nitride support membrane via spin coating (1st ramp: 500 rpm, 10 s; 2nd ramp: 3000 rpm, 30 s at an acceleration rate of 500 rpm s^−1^), achieving a particle density of ~1.2 particles per μm^2^. To minimize contamination issues arising from residual chemicals during exposure to the electron beam, the sample was treated by O_2_ plasma cleaning for 30 s. PVP (MW = 25,000) was purchased from Carl Roth GmbH & Co. KG, Germany. HAuCl_4_ ∙ 3H_2_O (99.9%), C_6_H_5_Na_3_O_7_·2H_2_O (≥99.5 %), and EtOH (≥99.9%) were obtained from Sigma-Aldrich Inc., MO, USA. DMF (≥99%) was obtained from Fluka, Honeywell Inc., NC, USA. Silicon nitride support membranes (30 nm, TA3003X-SF-HR) were purchased from Norcada Inc., AB, Canada. All reactants were used without further purification. Milli-Q water (18 MΩ cm^−1^) was used in all aqueous solutions, and all glassware was cleaned with aqua regia prior to usage.

### EELS, CL, and PINEM experiments

EELS and PINEM measurements were performed in STEM mode of a TEM instrument (JEM-2100F, JEOL Ltd., Japan) based on a custom-modified Schottky field emission source, with a selected electron-probe beam diameter of 1.5 nm. The spectral scans were recorded with an energy filtering and imaging device (CEFID, CEOS GmbH, Germany) equipped with a scintillator-coupled CMOS camera (TemCam-XF416ES, TVIPS GmbH, Germany) and synchronized by a universal scan generator (USG, TVIPS GmbH). EELS and PINEM spectra were acquired at binning resolutions of 15.6 meV and 16.6 meV, respectively. For EELS, a continuous electron beam was used with an initial energy spread of 0.5 eV (ZLP FHWM). For PINEM, the instrument was operated in an ultra-fast laser-triggered photoemission configuration enabling synchronous sample exposure by sub-picosecond electron probe and picosecond optical pump pulses. An amplified Ti:sapphire laser system (RegA, Coherent Inc., CA, USA) provided femtosecond pulses at a central photon energy of 1.55 eV (*λ* = 800 nm) and a spectral bandwidth of 65 meV (35-nm bandwidth) at a 600 kHz repetition rate. The optical pump pulses were dispersively stretched to a 3.4-ps pulse duration in a 19 cm bar of dense flint glass (SF6). The sample was excited under near-normal incidence (parallel to the electron beam) and a controllable polarization state with the light injected at an average power of ~4 mW and focused to a spot diameter of ~15 μm (corresponding to a maximum peak intensity of ~1.1 GW cm^−2^). Synchronous sub-picosecond electron-probe pulses were generated by photoemission from the Schottky field emitter using the second harmonic of the fundamental laser beam (for further details see ref. ^58^). The energy spread of the electron pulses was ~0.9 eV. PINEM and EELS spectra were acquired at integration time constants of 500 and 120 ms, respectively. The EELS spectra were deconvolved by the ZLP measured upon electron beam transmission through the silicon nitride support membrane using 20 iterations of a Richardson–Lucy (RL) algorithm^55^ implemented in the Hyperspy Python library^75^. Absolute EELS probabilities were obtained by normalizing the spectra to the integrated count rate measured upon electron beam transmission through the silicon nitride support membrane.

CL measurements were performed in an SEM instrument (FEI Quanta FEG 650, Thermo Fisher Scientific Inc., MA, USA) equipped with a Schottky field emission electron source and operated at an electron beam current of ~570 pA. CL emission was collected by a half-parabolic mirror covering a solid angle of 1.46 π sr above the sample plane and directed into an optical detection system for spectrally resolved CL analysis (SPARC Spectral, DELMIC BV, The Netherlands)^76^. The acquisition time for each spectrum was 350 ms, and the resolution of the spectrometer was on the order of 10 meV (as determined from the sharp emission lines of an argon calibration lamp). Secondary electron images were taken simultaneous to CL acquisition, and a software-controlled drift correction algorithm was applied at time intervals of 1 s to compensate for the effect of mechanical instabilities or electrostatic charging. The background luminescence from the silicon nitride support membrane was measured separately and subtracted from the raw CL data. The system response was calibrated, and absolute CL probabilities were obtained using the transition radiation (TR) spectrum measured upon 20 keV electron beam impact on the flat surface of a single-crystalline Al sample. The data were then normalized to the analytically calculated TR spectrum using the expression given in ref. ^2^ with optical material constants for the Al crystal derived from spectroscopic ellipsometry measurements.

### EELS and CL analysis

In the EELS spectra, the tip resonances were represented by a sum of Gaussians, reflecting the approximate shape of the ZLP. A background associated with (multiple) inelastic scattering was modeled by a Gaussian error function, rising from zero to a constant amplitude at energies >2.4 eV. Furthermore, a Gaussian centered between 2.3 and 2.9 eV was added to account for weak plasmonic contributions from the core and/or other tips. Another Gaussian bound to energies below 1.3 eV was used to account for a noise artefact of the RL algorithm in spectra with a low signal-to-noise ratio due to multiple scattering inside the nanostar. Plasmon resonances in CL were described by a sum of pseudo-Voigt distributions, capturing both their natural Lorentzian line shape and inhomogeneous broadening (i.e., due to electron beam-induced carbon contamination). A constant background was used to account for the emission of transition radiation or weak incoherent luminescence upon direct electron impact onto the nanostar. In our analysis procedure, the EELS and CL spectra were averaged first over segments of 10 × 10 pixels, and least-square minimization was applied to globally determine the central energy *E*_0_ and linewidth *γ* (FWHM) of the tip (and core) resonances. Subsequently, the amplitudes of the resonances were fitted to the spectrum at each electron beam position using the values retrieved for *E*_0_ and *γ*.

### PINEM analysis

The derivation of the electron-near-field coupling constant from the PINEM spectra was performed following a similar procedure as described in the supplementary information to ref. ^30^. Here, the initial electron energy distribution (i.e., prior to the near-field interaction) was modeled by a pseudo-Voigt profile with a Lorentzian-like contribution of 25% and an FWHM of 0.9 eV. Furthermore, we assumed a Gaussian distribution of the coupling constant $|\beta |$ with a standard deviation of ${{\Delta }}|\beta |/|\beta | = 0.2$ to account for residual spatial and temporal averaging in the strongly inhomogeneous optical near field. These effects arise from the finite probe size of the electron beam and the temporal profile of the optical pump pulses.

### BEM calculations

Numerical calculations were performed using the three-dimensional implementation of the BEM approach^52^ provided by the MNPBEM17 toolbox^53,54^ with optical constants for Au taken from tabulated optical data^60^. A triangular mesh was used to discretize the nanoparticle surface, with the meshing density gradually increasing from the tip shaft to the tip apex in order to account for highly localized charge accumulation. Electron-induced fields, as well as EELS and CL probabilities were calculated using built-in functions for electron beam excitation while assuming a finite beam width of 0.1 nm (see ref. ^54^ for details). For PINEM, a plane wave was assumed to be incident from above and polarized along the symmetry axis of the tip. The induced optical field was then calculated on a grid of points inside and outside the particle, extending up to half a wavelength above and below the tip apex. The stimulated coupling strength was obtained from the complex-valued plane-wave-induced optical field ${\mathbf{E}}^{\mathrm{L}}$ using the expression $(e/\hbar \omega ){\int} {{E}_z^{\mathrm{L}}(z)e^{ - i(\omega /v)z}{\mathrm{d}}z}$^22,25,37^. For electron trajectories intersecting with the nanoparticle, no integration points were placed closer than 0.25 nm to the particle surface to avoid numerical artefacts due to divergence of the fields at the Au/vacuum interface.
